# A kirigami-inspired soft gripper based on liquid crystal elastomer integrated with strain sensing

**DOI:** 10.1016/j.isci.2026.117188

**Published:** 2026-08-13

**Authors:** Chia-Yu Cho, You-Cheng Chen, Yao-Joe Yang

**Affiliations:** 1 Department of Mechanical Engineering, National Taiwan University, Taipei 10617, Taiwan

**Keywords:** Kirigami, miniaturized soft actuators, liquid crystal elastomers, self-sensing actuators, conductive composite, shape morphing

## Abstract

Soft robotic grippers are capable of handling objects with diverse shapes and sizes. However, achieving reliable grasping of delicate objects and sustaining prolonged holding with minimal energy input remains challenging. This study reports a miniaturized liquid crystal elastomer (LCE) soft gripper featuring a kirigami-inspired architecture, which is effective for capturing and securing delicate and slippery objects. The gripper consists of an LCE kirigami structure, which serves as the active material for generating deformation, and a conductive composite layer (CCL), which serves as a piezoresistive material for detecting deformations. In addition, the gripper opens its fingers when actuated, and closes its fingers for gripping objects when unactuated. Therefore, the device does not require any power when continuously holding objects. The gripper is also capable of detecting grasped object sizes enabled by the CCL. Demonstrations of grasping objects of various shapes, materials, and weights were also provided.

## Highlights

• A miniaturized kirigami soft gripper featuring self-sensing capability was proposed
• The gripper was realized using simple micromachining and vacuum filtration processes
• The self-sensing mechanism allows the device to detect the sizes of grasped objects
• The gripper is capable of continuously holding an object without consuming power

## Graphical abstract

**Figure:**
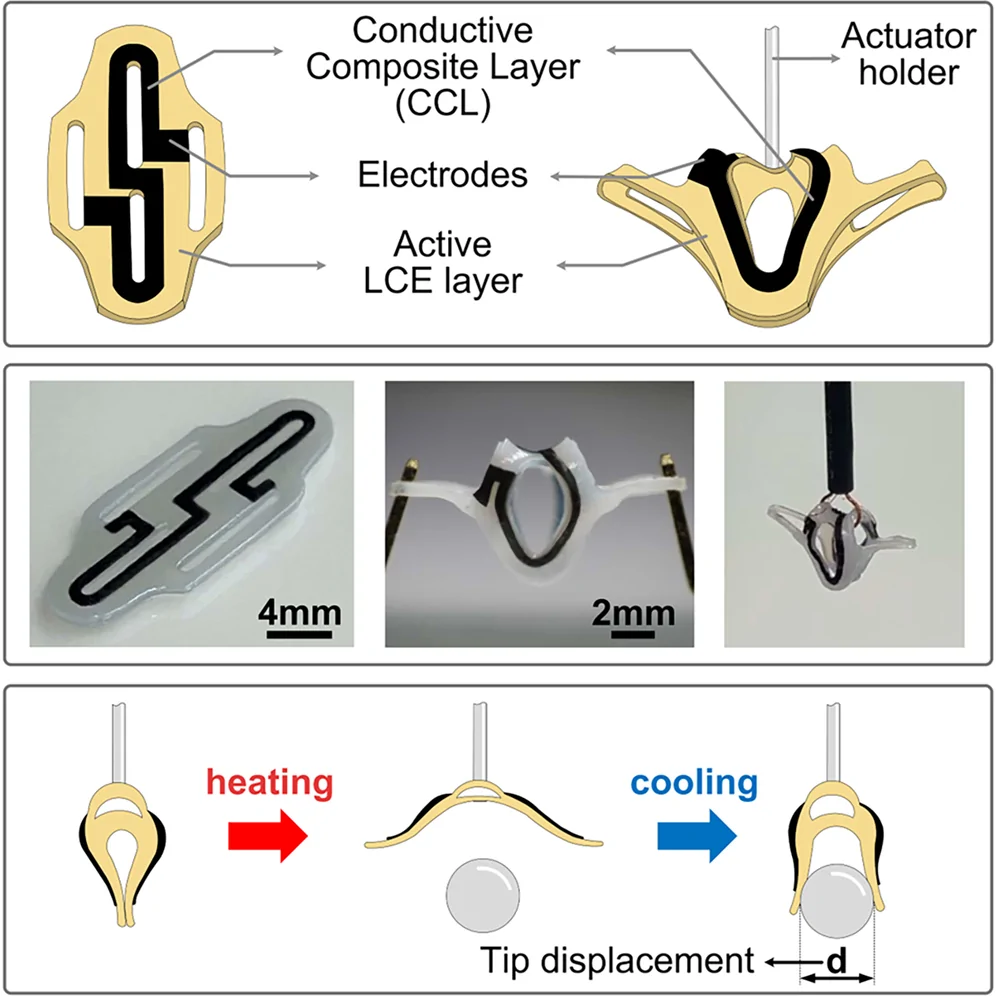

## Teaser

Mechanical engineering; Sensor system; Materials science

## Introduction

Soft actuators that exploit stimuli-responsive materials to achieve reversible deformation have gained significant interest for applications in soft robotics,^1^^,^^2^ sensors,^3^^,^^4^ artificial muscles,^5^^,^^6^ and biomedical devices.^7^ Various types of stimuli-responsive soft materials exhibiting different actuation behaviors, such as liquid crystal elastomers (LCEs),^8^ hydrogels,^9^ and dielectric elastomers,^10^ have been developed in recent decades to expand the performance landscape of soft actuators. Typically, these soft materials are capable of performing shape deformation triggered by external stimuli, such as temperature,^11^ light,^12^ magnetic fields,^13^ pH,^14^ and humidity.^15^

Among these materials, LCEs are particularly attractive due to their rapid response, phase transition-driven actuation, and high degree of programmability and controllability.^16^^,^^17^^,^^18^ Compared with other soft materials, LCEs exhibit relatively high elastic moduli and large reversible strains, making them suitable for soft actuators that require fast and substantial deformation. For instance, a self-healing LCE actuator was realized through a layered fabrication strategy based on dynamic bond exchange reactions in which actuation is driven by thermally induced fluid flow within embedded microchannels.^19^ In addition, bioinspired dry-spun LCE microfibers, which were successfully incorporated into skeletal-mimetic systems, exhibited excellent actuation speed, force output, and mechanical durability.^20^ Deng et al. reported a ring-shaped LCE actuator capable of continuous self-rotation under constant optical or thermal stimulation and demonstrated sustained locomotion across glass tubes, solid substrates, and liquid environments.^21^

In recent years, self-sensing LCE actuators with combined sensing and actuation capabilities have been proposed.^22^^,^^23^^,^^24^^,^^25^ Self-sensing actuators possess the inherent capacity to monitor their own condition while simultaneously performing their actuation functionality. Kent et al. reported LCE-based soft actuators incorporating liquid metal alloy-filled microfluidic channels that function as Joule heating elements.^26^ Ma et al. presented LCE-based self-sensing soft actuators which were realized through a simple magnetic printing process with liquid metal.^27^ Min et al. demonstrated intelligent self-sensing LCE actuators with enhanced mechanical strength and autonomous control capability by incorporating a composite of graphite and carbon black particles.^28^ Zhuo et al. developed a liquid crystal polymer foam-based self-sensing robotic system in which a highly porous open-cell structure formed via controlled solvent evaporation and ionic liquid incorporation enabled temperature sensing and actuation.^29^ In general, inducing deformation of shape-programmed LCE structures requires heating LCE structures to around 80 K above room temperature. In these studies, piezoresistive strain-sensing materials, such as liquid metals and ionic conductive polymers, typically suffer from temperature-induced drifts due to the intrinsic temperature dependence of their electrical resistance. Consequently, accurate strain sensing becomes difficult due to temperature elevation during the LCE actuation stage.

This study introduces a miniaturized, electrothermally actuated soft device with a kirigami-inspired architecture that serves as the mechanism for gripping action. The device is capable of generating large deformation while simultaneously providing intrinsic self-sensing of structural deformation. The proposed device comprises an LCE layer, which serves as the active material for generating deformation, and a conductive composite layer (CCL), which functions dually as an electrothermal heating element and piezoresistive sensing element. The CCL is mainly composed of carbon nanotubes (CNTs) and graphite microparticles. Techniques for reducing the temperature dependence of the CCL piezoresistive response were also employed to improve the device sensing performance.^30^^,^^31^^,^^32^^,^^33^^,^^34^ The device can be readily fabricated using straightforward micromachining techniques in combination with a novel vacuum filtration process, and is capable of estimating the size of the grasped objects.

## Results and discussion

### Design and actuation principle

**Figure 1:**
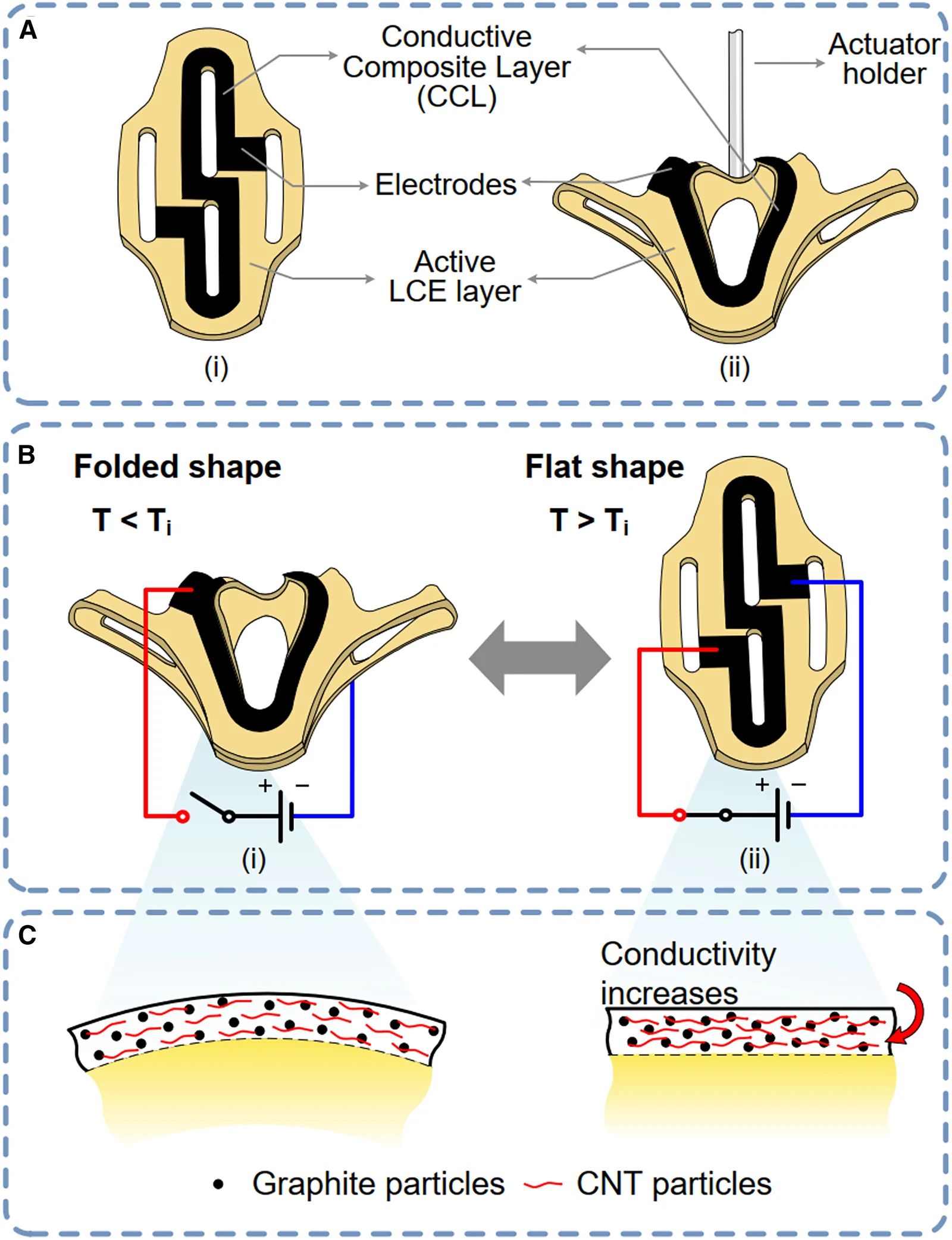
Design and operational principle of the proposed gripper (A) Schematic of the kirigami-inspired LCE actuator with self-sensing capability. (B) Schematic diagrams outlining the principle of shape-programmed actuation with electrothermal actuation. (C) Schematic of the self-sensing mechanism of the CCL.

Figure 1A(i) presents a schematic illustration of the proposed device prior to shape programming. The device comprises an active kirigami-based planar structure and an S-shaped CCL. The planar polymer structure is monolithically fabricated from LCE, while the CCL incorporates CNTs and graphite microparticles with varying weight percentages. As shown in Figure 1A(ii), when tensile forces are applied to the ends of the structure, out-of-plane deformation is induced because of the kirigami design, enabling the transformation from a two-dimensional (2D) patterned configuration into a three-dimensional (3D) grasping geometry. After a shape-programming process using ultraviolet (UV) illumination, the LCE structure is capable of reversibly switching between the 2D planar shape and the 3D grasping shape under thermal stimuli.

Figure 1B illustrates the operating principle of the device. Figure 1B(i) is a schematic of the structure after shape information has been encoded, corresponding to the folded (grasping) configuration. When voltage is applied across the two terminals of the S-shaped CCL, Joule heating causes the temperature elevation of the composite layer. Subsequently, the temperature of the LCE kirigami structure increases. When heated above the LC-to-isotropic phase transition temperature (*T*_*i*_), the LCE structure undergoes a rapid order-to-disorder transition, leading to programmed macroscopic deformation from the folded to the unfolded (planar) configuration, as shown in Figure 1B(ii). After the voltage is switched off, the LCE structure cools to room temperature and returns to its original shape (Figure 1B(i)) as a result of the reverse isotropic-to-LC phase transition. Notably, the capability of reversible 2D/3D transition of the kirigami structures enables the two fingers of the gripper to form a capsule shape when grasping an object. Compared to traditional soft grippers, the proposed gripper with this kirigami-based capsule shape is effective for capturing and securing delicate and slippery objects. In addition, the gripper opens its two fingers when actuated, while the gripper closes its fingers (i.e., in the capsule shape for gripping) when it is unactuated. In other words, when the gripper holds an object, it does not consume any energy. This operation mode is beneficial for the scenario where a gripper is required to hold an object for a long period of time. Furthermore, the S-shaped CCL deposited on the LCE structure functions not only as a resistive heater for LCE actuation but also provides self-sensing capability through the same pair of electrical leads used for electrothermal actuation, which will be described in the following subsection.

### Self-sensing capability with temperature compensation

Figure 1C schematically illustrates the self-sensing mechanism of the CCL. As shown in Figure 1C(i), when the kirigami-based LCE structure is heated and actuated, it deforms as a result of the LC-to-isotropic phase transition. The length of the S-shaped CCL deposited on the side surface of the LCE also decreases, as illustrated in Figure 1C(ii). Consequently, the CCL electrical resistance (measured across its two terminals) decreases because the CNTs and graphite microparticles in the CCL composite are brought closer together, thereby enhancing electrical conduction through the percolative network of the conductive fillers.

**Figure 2:**
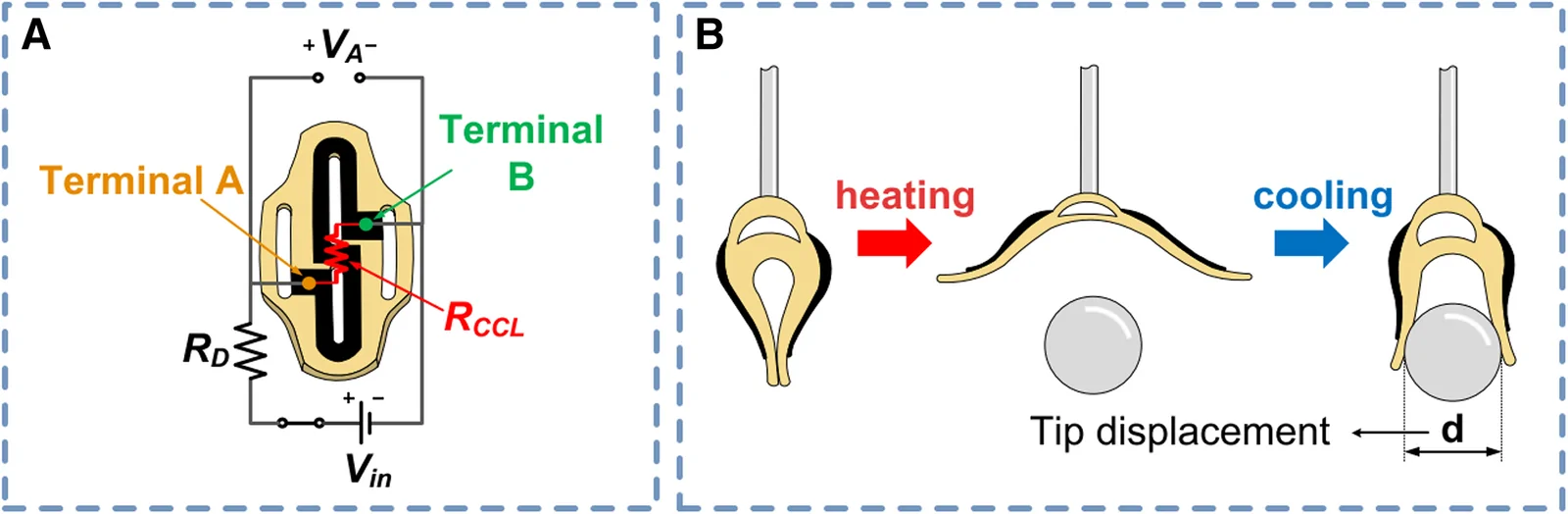
Self-sensing principle of the kirigami-inspired soft gripper (A) Circuit diagram for simultaneous actuation and sensation via the two terminals of the CCL. (B) Schematic of the operational principle of the gripper when touching an object.

Figure 2A illustrates a simplified circuit that enables simultaneous actuation and sensing through the same pair of CCL terminals (i.e., Terminals A and B). A divider resistor (*R*_*D*_) and the device (*R*_*CCL*_) are connected in series. A fixed voltage *V*_*in*_ is supplied across both resistors. The voltage drop across *R*_*CCL*_, denoted by *V*_*A*_, is the actuation voltage to heat up the LCE structure. Equation 1 presents the relationship between *V*_*A*_ and *R*_*CCL*_.

(Equation 1) $$V_{A}=\frac{R_{CCL}}{R_{D}+R_{CCL}}\;V_{in}$$

Rearranging Equation 1, we obtain:

(Equation 2) $$R_{CCL}=\frac{V_{A}}{V_{in}-V_{A}}\;R_{D}$$

in which *R*_*CCL*_ can be easily measured using an ADC circuit or a multimeter. Therefore, the strain of the LCE structure can be obtained by measuring *R*_*CCL*_.

However, during the actuation of the LCE kirigami structure, the CCL undergoes mechanical strain and also experiences an increase in temperature due to heating. Consequently, variations in *R*_*CCL*_ arise from two contributions: the piezoresistive response induced by mechanical strain and the temperature dependence of electrical resistance. To accurately estimate mechanical strain by measuring *R*_*CCL*_, the temperature coefficient of resistance (TCR) of the CCL must be minimized. Previous studies have demonstrated that a CCL composite with near-zero TCR can be obtained by appropriately adjusting the mass ratio of CNTs to graphite microparticles.^30^^,^^33^

The LCE kirigami structure integrated with a patterned CCL film enables simultaneous actuation and deformation detection within a single structure. Leveraging this hybrid capability, a miniaturized kirigami soft gripper is demonstrated, as shown in Figure 2B. The gripper can grasp objects in a wide range of sizes with arbitrary shapes. In addition, its self-sensing capability allows the gripper to determine the tip displacement (shown in the figure) from the *R*_*CCL*_, which varies with the size of the grasped object.

### Fabrication process

**Figure 3:**
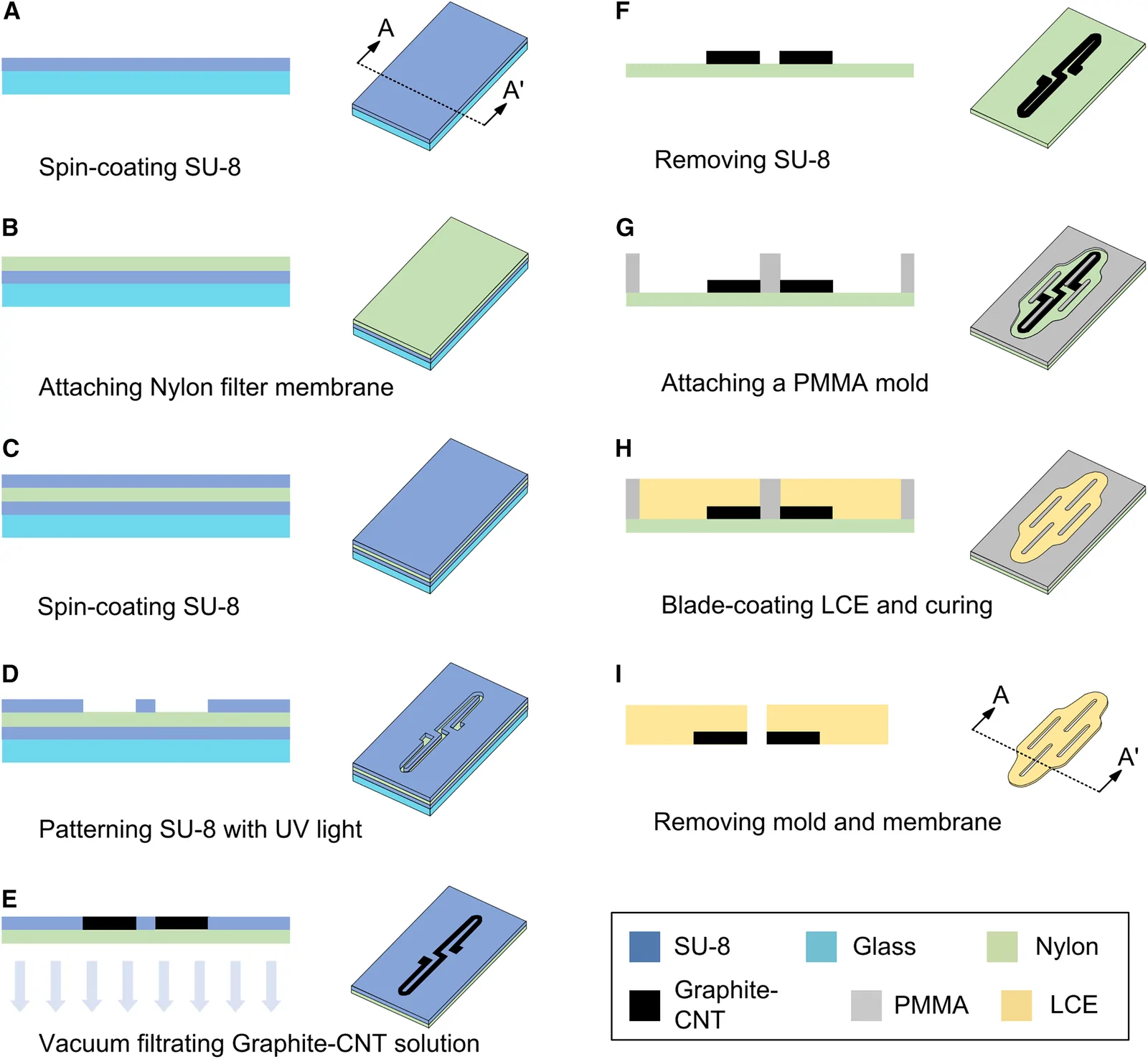
Fabrication process of the proposed self-sensing actuator

The fabrication procedure of the LCE kirigami structure with S-shaped CCL is illustrated in Figure 3. Figures 3A–3I describe the patterning process of the CCL using a standard soft lithography technique. First, a negative photoresist (SU-8 2050, MicroChem Corp.) was spin-coated onto a glass substrate at 1250 rpm, as shown in Figure 3A. This photoresist serves as a sacrificial layer. Subsequently, a nylon filter membrane (Rone Scientific Corp.) was placed on top of the photoresist layer (Figure 3B). As shown in Figure 3C, an additional SU-8 layer was spin-coated onto the filter membrane at 850 rpm. The SU-8 photoresist was patterned via a standard photolithography process following soft baking for 12 min, during which the filter membrane was released from the glass substrate in the development step (Figure 3D). A vacuum filtration process^30^ was then performed using a CNT-graphite-dispersed solution. As illustrated in Figure 3E, the CNT-graphite particles were deposited to fill the cavities of the patterned SU-8 mold. After removing the SU-8 mold using a PG remover (MicroChem Corp.), the patterned CCL remained deposited on the filter membrane (Figure 3F). Subsequently, a laser-cut poly(methyl methacrylate) (PMMA) mold with predefined kirigami patterns was assembled on top of the filter membrane (Figure 3G). Then, the LCE prepolymer was blade-coated into the mold (Figure 3H). The coated prepolymer was cured at 85 °C for 24 h to ensure complete solvent evaporation. Finally, the patterned LCE structure integrated with the CCL was peeled from the filter membrane and released from the PMMA mold, as shown in Figure 3I. The preparation of the CNT-graphite-dispersed solution and the LCE prepolymer solution will be described in the STAR Methods.

**Figure 4:**
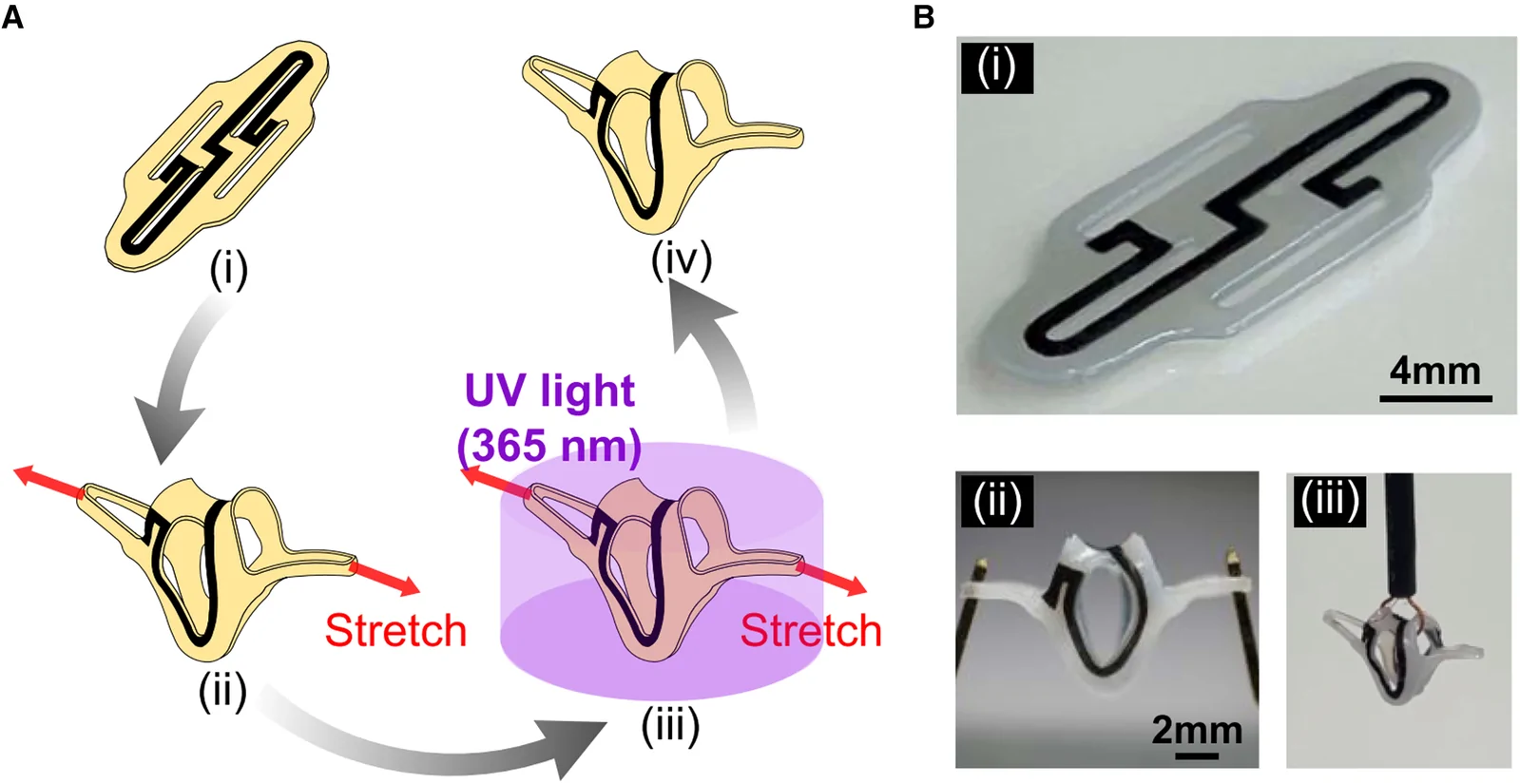
The shape programming process and fabricated devices (A) Shape programming process of the LCE actuator achieved through mechanical stretching and photo-cross-linking. (B) The fabricated kirigami soft actuator (i) before shape programming, (ii) after shape programming, and (iii) assembled with a holder.

Figure 4A illustrates the shape programming process of the LCE achieved through mechanical stretching and photo-cross-linking. To ensure that the mechanical stretching process is well-controlled, we implemented a stretching fixture consisting of a manual linear translation stage and two pin headers (Figure S1). As shown in Figures 4A(i) and 4A(ii), by pulling on the ends of the structure, which induces out-of-plane bending due to the proposed kirigami design, the structure transformed from a 2D pattern into a 3D grasping shape. This out-of-plane bending mechanically aligned LC mesogens in the structure, leading to the formation of local monodomains with ordered LC phases. Then, the structure was subjected to UV irradiation to lock in the ordered alignment of LC mesogens, completing the second cross-linking stage (Figure 4A(iii)). Figure 4A(iv) shows the LCE structure after the shape programming process. Figures 4B(i) and 4B(ii) present the pictures of the fabricated device before and after the shape programming, respectively, while Figure 4B(iii) shows the device assembled with a holder.

### Device characterization and demonstration

**Figure 5:**
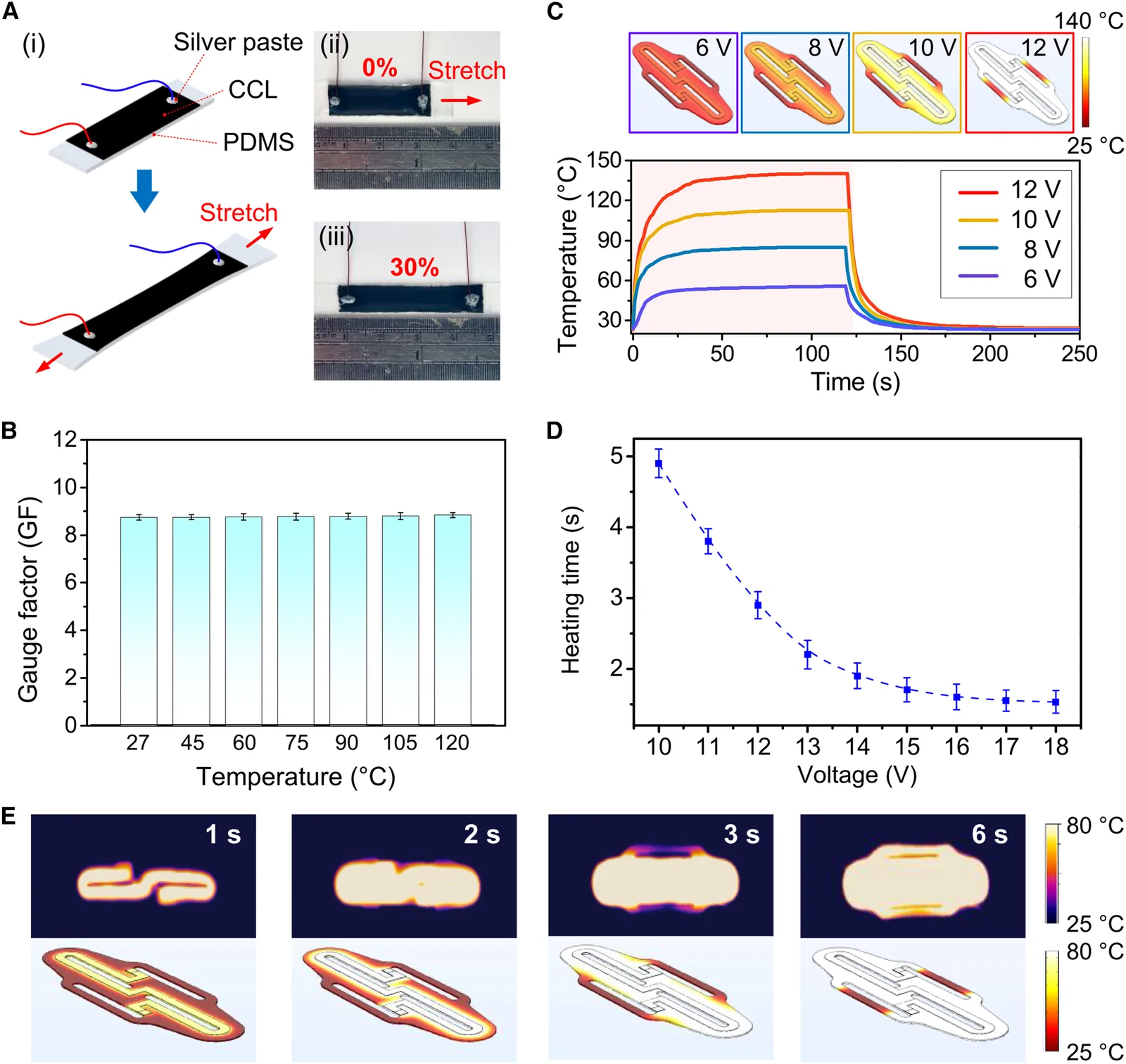
CCL sensing performance and LCE thermal response (A) (i) Experimental configuration for characterizing the relative resistance change of CCLs under tensile strain at various temperatures. Photographs of the sample before (ii) and after (iii) stretching of *ε* = 30%. (B) GF values of a CCL (1:10 CNT-graphite ratio) as a function of temperature. Data are represented as mean (*n* = 10), with error bars indicating the minimum-to-maximum range. (C) Transient temperature response of the device at different actuating voltages. FEA-simulated steady-state temperature distributions were also included. Both heating and cooling times are sufficient for device temperatures to reach a steady state. (D) Required heating duration to reach above the phase transition temperature *T*_*i*_ (70 °C) vs. applied voltages. Data are represented as mean (*n* = 10), with error bars indicating the minimum-to-maximum range. (E) Infrared camera images of temperature distribution on the device top surface under Joule heating of 12 V at different points of time (i.e., 1 s, 2 s, 3 s, and 6 s). The FEA simulated results were consistent with the measured results.

To achieve optimized piezoresistive sensing performance with temperature compensation, the TCRs of CCLs with different compositions were characterized.^30^^,^^31^^,^^32^^,^^33^ These results are presented in Figure S2. The composite with a 1:10 CNT-graphite ratio exhibits the lowest TCR and is therefore selected for implementation in the proposed devices. Figure 5A illustrates the schematic for measuring the relative resistance change of the CCL under stretching at different temperatures. Prior to the measurement, the PDMS thin film with the CNT-graphite layer was machined into a strip with a length of 40 mm and a width of 17 mm. Subsequently, the strip sample was subjected to tensile loading via an externally applied force. Concurrently, a constant voltage was applied across the two terminals of the CCL, inducing Joule heating while enabling simultaneous measurement of the resistance change during deformation. The gauge factors (GFs) of the CCL composite measured at different temperatures are summarized in Figure 5B. The results indicate that the variations of GF values are very small (<1%) across a broad temperature range from 27 °C to 120 °C, and further confirm that the temperature-induced effects on strain sensing are negligible due to the effectively minimized TCR of the CCL composite. Each data point on these curves is the average result of 10 measurements, and the error bars of each data point indicate the measured maximum and minimum values.

As shown in Figure 5C, the measured temperature responses of the LCE device by Joule heating vary with the applied voltage and actuation time. Finite element analysis (FEA) simulation results of steady-state temperature distribution of different heating voltages, obtained using COMSOL Multiphysics (Burlington, MA, USA), are also shown in the figure. The device exhibits a rapid temperature rise with increasing applied voltage and reaches a steady state within 30 s. The steady-state temperatures at voltages of 6–12 V are 58 °C, 85 °C, 112 °C, and 140 °C. The heating time required to reach the steady-state temperature from room temperature remains nearly constant across different applied voltages, indicating that the rise time is largely voltage independent. By contrast, the steady-state temperature varies with voltage and increases monotonically with the applied voltage. When the applied voltage exceeds 12 V, excessive temperature elevation may damage the structural integrity and functional performance of the device.

It is possible to speed up the heating process with a short pulse of high voltage to achieve the phase transition temperature (> *T*_*i*_) while avoiding excessive temperature elevation. Therefore, a series of transient measurements with short heating times was performed (as shown in Figure S3) to study the relationship between the required heating times for exceeding *T*_*i*_ (70 °C) vs. the applied voltages. The results are summarized in Figure 5D. Obviously, the higher the applied voltage, the shorter the required heating time. In this work, an actuation voltage of 12 V was selected, as it represents a practical compromise between a relatively low driving voltage and a sufficiently short heating time for LCE-based devices. Each data point is the average value of 10 measurements, and the error bars of each data point indicate the measured maximum and minimum values.

Figure 5E and Video S1 show infrared camera images captured using an infrared thermal camera (PI 640i, Optris GmbH, Berlin, Germany), further revealing the temperature distribution on the LCE structure after heating at 12 V at different times (1 s, 2 s, 3 s, and 6 s). FEA simulation results are also shown in the figure. Video S2 also demonstrates that the LCE structure exhibits a relatively uniform temperature distribution after heating for 6 s. The device temperature in most regions is greater than the LCE phase transition temperature (*T*_*i*_). This result confirms that Joule heating from the CCL can effectively heat the active LCE region and trigger the phase transition for global shape deformation.

Video S1. Device temperature distribution under Joule heating (prior to shape programming), related to Figure 5

Video S2. Gripper operation: opening and closing, related to Figure 5

**Figure 6:**
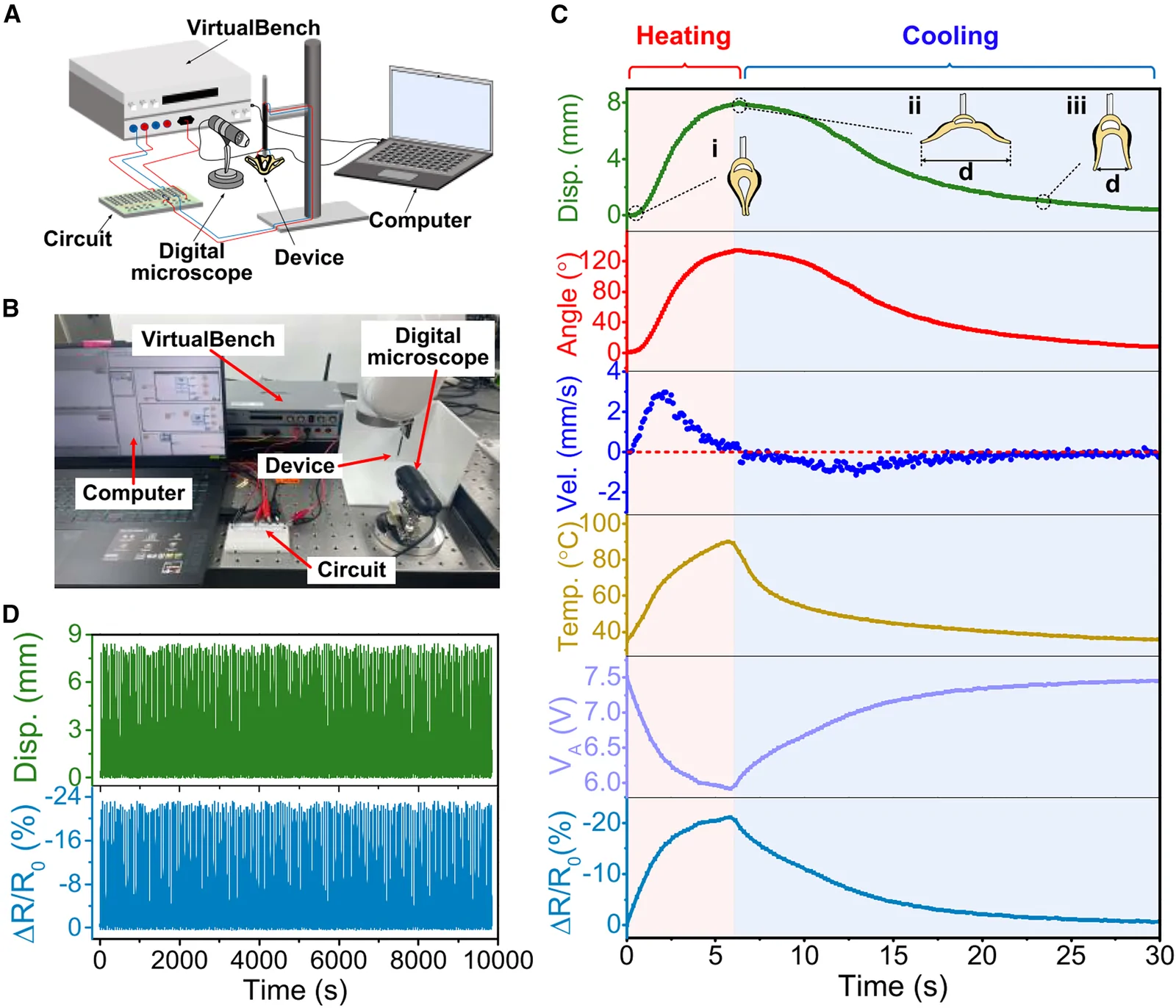
The operation and performance of the kirigami-based soft gripper (A) Schematic diagram of the experimental setup for measuring the resistance change of the device. (B) Photograph of the complete experimental setup. (C) Transient responses of the temperature, displacement, angle, tip velocity, resulting voltage across CCL (*V*_*A*_), and resistance at the given voltage for an actuation cycle. (D) Transient responses of tip displacement and resistance for 200 actuation cycles.

Figure 6A illustrates the schematic diagram of the experimental setup used to measure the transient resistance of the CCL (*R*_*CCL*_) during electrothermal actuation, while Figure 6B shows the photograph of the complete experimental setup. The corresponding circuit configuration enables simultaneous actuation and sensing through the two terminals of the S-shaped CCL. The actuation voltage was supplied through a power MOSFET switch. A data acquisition system (VirtualBench NI VB-8012, National Instruments Corp.) was employed to provide the input signal (*V*_*in*_) and to acquire the sensing voltage (*V*_*A*_). Figure 6C illustrates the transient responses obtained from a single actuation cycle of the LCE actuator, in which a 12 V step voltage is applied for 6 s, followed by a 40 s natural cooling phase. The insets shown in the figure illustrate the gripper configurations at different stages. Upon application of a step voltage, the device temperature rapidly rises to approximately 90 °C, triggering deformation of the LCE structure from the folded (i.e., the programmed shape) configuration to the unfolded shape via the LC-isotropic phase transition. The tip displacement markedly increases once the temperature approaches or exceeds 70 °C. After the applied voltage is reduced to 3 V, the device cools naturally to room temperature (≈25 °C) and returns to the folded (grasping) configuration. The device temperature was monitored using a thermocouple (NI USB-9213, National Instruments Corp.). The variation in *R*_*CCL*_ can be evaluated by *V*_*A*_, which was measured by the data acquisition system. The tip displacement and tip opening angle of the gripper were obtained by analyzing the recorded videos with the open-source video analysis software Tracker (Open Source Physics Projects). Obviously, *R*_*CCL*_ closely tracks the deformation (i.e., the tip displacement) of the LCE structure. The fabricated device exhibits an initial CCL resistance (*R*_0_) of 1.12 kΩ. When actuated at 12 V, *R*_*CCL*_ decreases from 1.12 kΩ to 785 Ω. In addition, the tip velocity was evaluated from the measured tip displacement. The tip velocity was approximately 3 mm s^−1^ during the opening process and approximately −1.2 mm s^−1^ during the closing process. The negative value corresponds to the closing direction of the gripper tip. In addition, the gripper reached an opening angle of approximately 135° within 6 s at an applied voltage of 12 V. After the applied voltage was reduced to 3 V, the gripper gradually closed during the natural cooling process and returned to the folded configuration within 24 s.

The repeatability test was also conducted under identical actuation conditions, as shown in Figure 6D. After more than 200 actuation cycles, only negligible variations in gripper tip displacement and relative resistance change were observed. The variation between each cycle is approximately 3.3% for the tip displacement and 2.5% for the relative resistance change. These results demonstrate the device’s excellent stability and reversibility.

**Figure 7:**
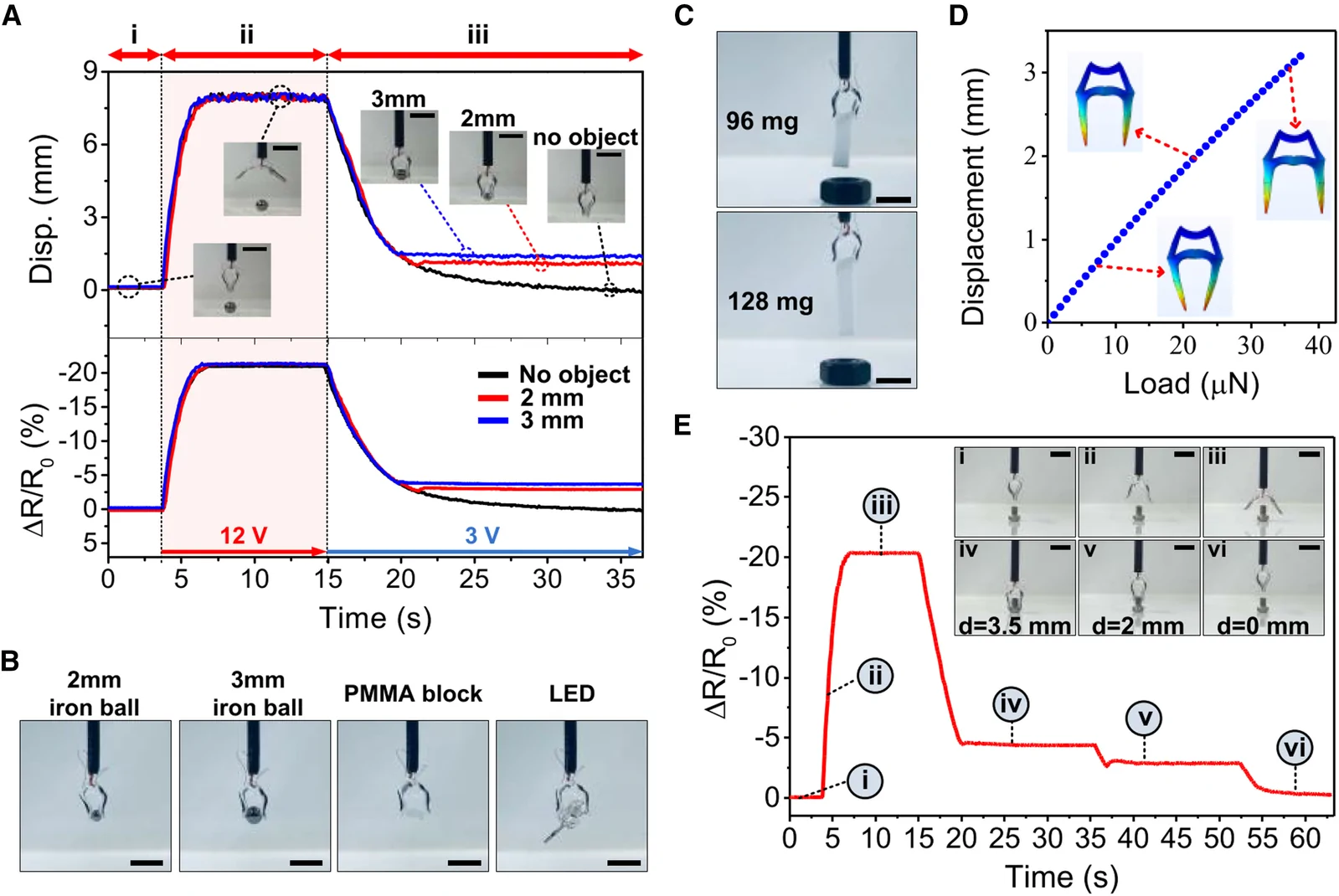
Object gripping demonstration of the kirigami-based soft gripper (A) Transient responses of resistance when the gripper grasps an object. A series of snapshot images are also shown. The inset shows pictures of the actuator at different stages. (B) The kirigami-based soft gripper grasps different objects, including iron balls (diameters of 2 mm and 3 mm), a PMMA block, and an LED. Scale bars, 6 mm. (C) The soft gripper grasps PMMA rectangular bars of different weights (96 mg and 128 mg; the cross section of the samples is 3 mm × 3 mm). Scale bars, 6 mm. (D) FEA-simulated relationship between the gripping force and the finger opening. (E) The corresponding resistance changes at different gripping positions along the object.

Figure 7A shows the tip displacement and resistance responses of the kirigami-based soft gripper. Each figure includes three curves corresponding to the sizes of grasped objects (i.e., 2 mm, 3 mm, and no object). These curves clearly show that the integrated self-sensing mechanism enables the device to estimate the size of the grasped objects. Three distinct stages for each grasping process are also annotated in the figure. In Stage i, the gripper is closed (i.e., the closed state) in the absence of an applied voltage. In Stage ii, a heating voltage of 12 V is supplied to the S-shaped CCL. The gripper starts to deform due to the LCE phase transition caused by temperature elevation. The tip displacement of the gripper’s fingers increases rapidly and subsequently stabilizes, indicating that the gripper has reached a fully open state. During this finger opening process, the deformation of the LCE structure induces the strain of the S-shaped CCL, leading to the decrease in CCL resistance. In Stage iii, the applied voltage is reduced from 12 V to 3 V, and the gripper starts to cool down. Therefore, the gripper gradually retreats to its original shape (i.e., the closed state), and starts to grasp an object. Simultaneously, the CCL resistance increases as the gripper retreats to its closed shape. By using the simple circuit shown in Figure 2A, the CCL resistance can be easily measured with the applied voltage of 3 V in Stage iii. The final steady-state resistance value correlates with the closing displacement of the gripper tips, which is the size of the grasped object. This steady-state resistance value decreases as the diameter of the grasped object increases. Figure 7B and Videos S3, S4, S5, and S6 show that the kirigami-based soft gripper is also capable of grasping objects of various shapes, including two iron balls, a PMMA block, and an LED. Figure 7C and Videos S7, S8, and S9 demonstrate that the gripper successfully grasps PMMA rectangular bars of different weights (The cross section of the samples is 3 mm × 3 mm). Based on the measurement, the maximum graspable weight is approximately 128 mg, which is about 8.5 times its own weight (15 mg).

Video S3. Object grasping demonstration (2 mm iron ball), related to Figure 7

Video S4. Object grasping demonstration (3 mm iron ball), related to Figure 7

Video S5. Object grasping demonstration (PMMA block), related to Figure 7

Video S6. Object grasping demonstration (LED), related to Figure 7

Video S7. Grasping a PMMA bar (3 × 3 × 3 mm^3^, 32 mg), related to Figure 7

Video S8. Grasping a PMMA bar (3 × 3 × 9 mm^3^, 96 mg), related to Figure 7

Video S9. Grasping a PMMA bar (3 × 3 × 12 mm^3^, 128 mg), related to Figure 7

The proposed gripper maintains its folded configuration (i.e., the grasping shape) when it is unactuated. At this moment, if the gripper does not grasp any object, it is in its original folded configuration (i.e., its original shape) programmed by the shape programming process. However, when the gripper grasps and holds an object, the final shape of the gripper differs from its original shape because the grasped object prevents the gripper from retreating to its original shape. The shape difference between the gripper’s final shape and original shape induces internal stress inside the gripper structure and can be used to estimate the gripping force. We constructed a three-dimensional FEA model of an unactuated gripper (i.e., original shape), and calculated the deformation of the gripper by applying different force loads on the regions of the fingertips (COMSOL Multiphysics, Burlington, MA, USA). Figure S4 presents the deformed shapes of various loads simulated by the FEA solver. The relationship between the gripping force and the finger opening is shown in Figure 7D.

Figure 7E demonstrates the potential application of the kirigami-based soft gripper for object size detection. A 20% reduction in resistance is observed as the gripper transitions from the fully closed state (stage i) to the fully open state (stage iii). Then, the applied voltage is reduced to 3 V, the cooling process triggers the closure of the gripper to facilitate object acquisition. Compared with the fully closed state, the measured resistance drops of about 4.2% for the screw head (3.5 mm, Stage iv), and 2.7% for the threaded shank of the screw (2 mm, Stage v), respectively. Such responsive characteristics validate the feasibility of employing this gripper for identifying the dimensions of unknown objects.

### Conclusions

In this study, we proposed a miniaturized kirigami-inspired soft gripper driven by electrothermal actuation capable of large-amplitude deformation while inherently providing self-sensing for monitoring structural deformation. The device consists of an active, kirigami-inspired planar structure integrated with an S-shaped CCL. Shape information is programmed into the LCE structure through a shape memory process. The actuation of the LCE is achieved via Joule heating generated by the CCL, while deformation is simultaneously detected through its piezoresistive response. The TCR of the CCL was effectively minimized to accurately estimate the mechanical strain response of the CCL. The results show that the integrated self-sensing mechanism allows the device to distinguish between grasped objects of different sizes. With the proposed kirigami design, the proposed gripper is capable of maintaining a grasp of an object without continuously heating the LCE. The proposed strategy, with combined actuating and sensing functionalities, offers strong potential for the development of smart soft robotic systems and other intelligent devices.

### Limitations of the study

The present study did not examine the influence of the CCL shapes on the LCE actuation, and this issue should be explored further in future research. In addition, although the present study has demonstrated the object grasping and object size detection capabilities of the gripper, the graspable weight range is relatively difficult to estimate because it may be affected by the object shapes and friction on contact surfaces. Moreover, the gripper still relies on external wiring and a separate power supply, which constrain its use in certain practical applications. Also, the CCL sensing resolution for grasping objects of smaller sizes still requires further investigation. Future efforts may overcome these limitations through the development of grippers integrated with embedded systems, such as onboard power sources and control circuits.

## Resource availability

### Lead contact

Further information and requests for resources should be directed to and will be fulfilled by the lead contact, Yao-Joe Yang (yjy@ntu.edu.tw).

### Materials availability

This study is based on existing materials and does not produce new materials.

### Data and code availability

• All data reported in this paper will be shared by the lead contact upon request.
• This article does not report any original code.
• Any additional information required to reanalyze the data reported in this article is available from the lead contact upon request.

## Acknowledgments

This work was supported in part by the 10.13039/100020595National Science and Technology Council, Taiwan, R.O.C. (contract no. 112-2221-E-002 -147 -MY3).

## Author contributions

C.-Y.C.: data curation, formal analysis, investigation, methodology, validation, visualization, and writing – original draft. Y.-C.C.: data curation, formal analysis, and software. Y.-J.Y.: conceptualization, formal analysis, investigation, project administration, resources, supervision, validation, writing – original draft, and writing – review and editing.

## Declaration of interests

The authors declare that they have no known competing financial interests or personal relationships that could have appeared to influence the work reported in this paper.

## STAR★Methods

### Key resources table

**Table:** 

REAGENT or RESOURCE	SOURCE	IDENTIFIER
**Chemicals, peptides, and recombinant proteins**		

1,4-bis-[4-(3-acryloyloxypropyloxy)benzoyloxy]-2-methylbenzene (RM257)	Wilshire Technologies	N/A
2,2’-(ethylenedioxy)diethanethiol (EDDET)	Sigma-Aldrich	Cat# 465178
Pentaerythritol tetrakis(3-mercaptopropionate) (PETMP)	Sigma-Aldrich	Cat# 381462
2-hydroxy-4-(2-hydroxyethoxy)-2-methylpropiophenone (HHMP)	Sigma-Aldrich	Cat# 410896
Catalyst dipropylamine (DPA)	Alfa Aesar	N/A
Toluene	Echo Chemical Co., Ltd.	A-A-59107
DI water	Echo Chemical Co., Ltd.	Cat# 112431034
Ethyl Alcohol (95%, v/v)	Echo Chemical Co., Ltd.	N/A
Polydimethylsiloxane (PDMS)	Dow Inc.	SYLGARD™ 184 Silicone Elastomer Kit
SU-8 photoresist 2050	MicroChem Corp.	SU-8 2050
SU-8 developer	MicroChem Corp.	https://www.microchem.co
PG remover	MicroChem Corp.	https://www.microchem.co
Isopropyl Alcohol	Echo Chemical Co., Ltd.	Cat# D304649
Poly(methyl methacrylate) (PMMA)	Wu-Mei Acrylic Industrial Co.	N/A
Nylon filter membrane	Rone Scientific Corp.	N/A
CNTs	Golden Innovation Business Co., Ltd.	AC tube-100L-TSMO015
Graphite microparticles	Homytech Global Co., Ltd.	G-H6-995
Silver paste	Rui-Long Science Research Market Co., Ltd.	Cat# RLA2K40001

**Software and algorithms**		

COMSOL Multiphysics	COMSOL Multiphysics, Burlington	https://www.comsol.com
Origin	OriginLab	https://www.originlab.com
Tracker	Open Source Physics Projects	https://opensourcephysics.github.io/tracker-website

**Other**		

VirtualBench NI VB-8012	National Instruments Corp.	https://www.ni.com
Infrared thermal camera	Optris GmbH	https://optris.com
Sonicator	Qsonica, LLC	https://www.sonicator.com
Thermocouple input module	National Instruments Corp.	https://www.ni.com
UV source	Hsin An Instrument Co., Ltd.	CU-UVGL28
Spin coater	Hsin An Instrument Co., Ltd.	N/A
Laser cutter	TAIWAN 3 Axle Technology Co.,Ltd.	SU3-6040-100W
Mask Aligner	M&R Nano Technology Co., Ltd	EVG 620
Vacuum pump	ULVAC, Inc.	GLD-101
Oven	Yuan Tuo Technology Co., Ltd.	DH400
Hotplate	Chemist Scientific Corp.	HP-330DA

### Method details

#### Preparation of the CNT–graphite-dispersed solution

First, 3 mg of CNTs (AC tube-100L-TSMO015, Golden Innovation Business Co., Ltd.) and 30 mg of graphite microparticles (G-H6-995, Homytech Global Co., Ltd.) were dispersed in 330 mL of an alcohol–water mixture with a 7:3 volume ratio. The mixture was subjected to ultrasonication for 1 h using a sonicator (Q700, Qsonica, LLC), with intermittent pauses of 2 s after every 15 s of sonication.

#### Preparation of the LCE prepolymer solution

LC mesogen RM257 (95%, 2.0 g, Wilshire Technologies, Princeton, NJ, USA) was dissolved in 0.6 g of toluene by heating at 85 °C for 3 min. After the solution was cooled to room temperature, the photoinitiator HHMP (98%, 0.012 g, Sigma-Aldrich, San Luis, MO, USA), chain extender EDDET (95%, 0.423 g, Sigma-Aldrich, San Luis, MO, USA), tetrafunctional thiol cross-linker PETMP (95%, 0.094 g, Sigma-Aldrich, San Luis, MO, USA), and catalyst DPA (99%, 0.288 g, Alfa Aesar, Haverhill, MA, USA) were sequentially added. The resulting mixture formed a loosely cross-linked LCE network via a thiol–Michael addition reaction. After thorough mixing and degassing, the resulting prepolymer was ready for the subsequent molding step.

#### Fabrication of SU-8 mold

A negative photoresist (SU-8 2050, MicroChem Corp.) was spin coated onto a glass substrate at 1250 rpm for 60 s using a spin coater (Hsin An Instrument Co., Ltd.). This photoresist layer served as a sacrificial layer. The coated glass substrate was soft baked on a hotplate (HP-330DA, Chemist Scientific Corp.) at 65 °C for 5 min. Subsequently, a nylon filter membrane (Rone Scientific Corp., 47 mm in diameter, pore size 0.8 μm) was placed on top of the photoresist layer. An additional SU-8 layer was spin coated onto the nylon filter membrane at 850 rpm for 100 s. The SU-8 photoresist was then soft baked in two sequential stages, first at 65 °C for 12 min and then at 95 °C for 15 min. The coated sample was aligned with a photomask and then exposed under UV irradiation using a mask aligner (EVG 620, M&R Nano Technology Co., Ltd.) for 30 s. The UV exposure wavelength was 365 nm, and the intensity was 23 mW cm^−2^. After UV exposure, the sample was under post exposure bake in two sequential stages, first at 65 °C for 5 min and then at 95 °C for 12 min. Subsequently, the sample was developed in the SU-8 developer (MicroChem Corp.) for 5 min under continuous agitation. During the development step, the glass substrate was also detached from the nylon filter membrane because the sacrificial layer was resolved. Finally, the nylon filter membrane with the patterned SU-8 mold was rinsed with isopropyl alcohol (IPA, Echo Chemical Co., Ltd.) for 10 s and dried using nitrogen gas.

#### CCL deposition process

The CNT–graphite-dispersed solution was poured onto the patterned SU-8 mold and held for 2 min, followed by vacuum filtration for 40 min using a vacuum pump (GLD-101, ULVAC Taiwan Inc.) to deposit the CNT–graphite composite within the patterned regions. Subsequently, the deposited CNT–graphite membrane was heat-treated in an oven (DH400, Yuan Tuo Technology Co., Ltd.) at 90 °C for 30 min. The SU-8 mold was then removed by immersion in PG remover (MicroChem Corp.) at 70 °C for 30 min. After removing the SU-8 mold, the patterned CCL was rinsed with isopropyl alcohol (IPA, Echo Chemical Co., Ltd.) for 10 s and then dried using nitrogen gas. The nylon filter membrane patterned with CCL was ready for the following LCE molding process.

#### LCE molding process

By using a laser cutter (SU3-6040-100W, TAIWAN 3 Axle Technology Co., Ltd), A poly(methyl methacrylate) (PMMA) mold with predefined kirigami patterns was fabricated from a 0.5 mm thick PMMA sheet. The laser power is 70 W, and the cutting speed is 30 mm s^−1^. Subsequently, the fabricated PMMA mold was placed on top of a nylon filter membrane. Both the PMMA mold and the nylon filter membrane are fixed by a few binder (dovetail) clips. The LCE prepolymer was then poured into the PMMA mold. After removing the excess LCE prepolymer above the PMMA mold surface by using a blade, the LCE prepolymer (with the PMMA mold) was placed in an oven (DH400, Yuan Tuo Technology Co., Ltd.) and cured at 85 °C for 24 h to ensure complete solvent evaporation. Finally, the patterned LCE structure integrated with the CCL was peeled from the nylon filter membrane and released from the PMMA mold.

#### LCE mechanical stretching

To ensure sample consistency during mechanical stretching, we implemented a stretching fixture consisting of a manual linear translation stage and two pin headers, as shown in Figure S1. The ends of the LCE kirigami structure clasp on the pins of the pin headers during the stretching process. The linear stage precisely stretches the LCE kirigami structure to a predefined strain of 33%, with the stretching direction aligned with the main axis of the kirigami structure to reduce device-to-device variation.

#### LCE photopolymerization

After mechanically stretched and maintained at the predefined strain (33%) on the stretching fixture, the LCE kirigami structure was subjected to UV irradiation for 8 h by using a UV source (CU-UVGL28, Hsin An Instrument Co., Ltd.) to lock in the ordered alignment of LC mesogens, completing the second cross-linking stage. The UV wavelength is 365 nm, and the irradiation intensity is 10 mW cm^−2^.

#### Device assembly

After the shape programming process, electrical leads were glued to the CCL contacts (i.e., Terminals A and B) of the LCE kirigami device using silver paste (Rui-Long Science Research Market Co., Ltd.). The LCE device was subsequently used for electrothermal actuation and resistance measurements.

#### FEA simulation of grippers

FEA simulations of gripper deformation were performed using COMSOL Multiphysics (Burlington, MA, USA). The material properties used in the simulations are listed in Table S1. For the gripping force simulations, “Solid Mechanics” module with “Linear Elastic Material” was employed. The mesh contained 92,195 tetrahedral elements and 41,052 triangular elements. Figure S4 presents the FEA-simulated results of gripper deformation with different force loads (0–2.5 Pa) applied on the regions of finger tips. The applied force loads on the gripper fingers can be considered as the contact force exerted by the grasped object. The gripping force increases with the finger opening, which is proportional to the size of the grasped object.

For the thermoelectric analysis for estimating the device temperature distribution by Joule heating, “Electric Currents” and “Heat Transfer in Solids” modules were employed, and the “Joule Heating” was selected as multiphysics coupling. The mesh contained 97,067 tetrahedral elements and 42,225 triangular elements. The initial temperature was set to 293.15 K, and the other settings was set as default. To better resolve the temperature variations in regions with different thicknesses, a finer mesh was applied to the thinner regions. A reasonable mesh density not only reduces computational resource consumption and improves calculation efficiency but also ensures sufficient calculation accuracy.

#### Data analysis

The quantitative data of tip displacement and opening angle were extracted from the recorded movie with video analysis software (Tracker).

### Quantification and statistical analysis

Tracker and Origin 2018 were used for measurements and statistical analysis. For repeated measurements shown in the Results section, each data point is the average result of 10 measurements, and the error bars of each data point indicate the measured maximum and minimum values. In addition, the surface temperature distributions were visualized by an infrared thermal imager (PI 640i, Optris GmbH, Berlin, Germany) The captured thermal images were processed using the software provided by the vendor of the thermal imager (Optris PIX Connect, Optris GmbH, Berlin, Germany).
