# Supplementary material for: A kirigami-inspired soft gripper based on liquid crystal elastomer integrated with strain sensing
Source: iScience. 2026 Aug 13;29(9):117188. doi: 10.1016/j.isci.2026.117188 (PMC13495404; doi:10.1016/j.isci.2026.117188)
Supplement: Supplementary file 1 [file mmc1.pdf]

iScience, Volume *29*

Supplemental information

A kirigami-inspired soft gripper based on liquid crystal elastomer integrated with strain sensing

Chia-Yu Cho, You-Cheng Chen, and Yao-Joe Yang

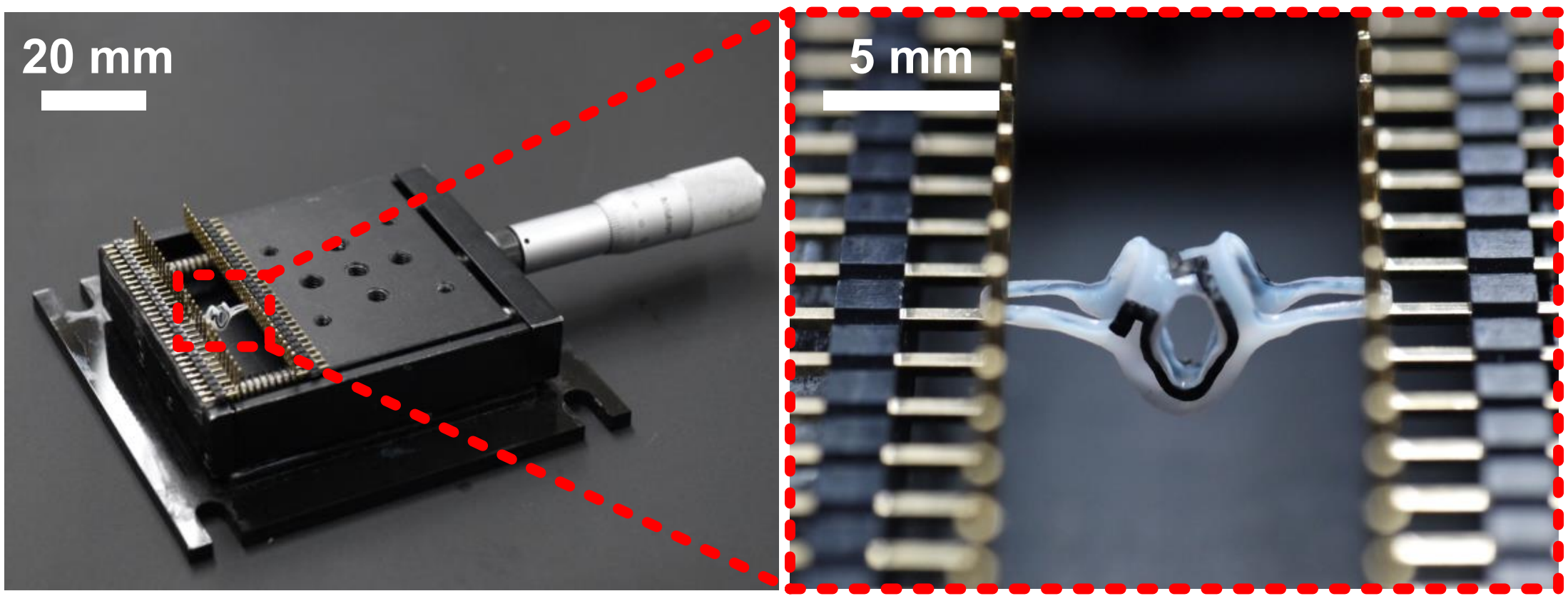

Figure S1. Pictures of the stretching fixture consisting of a manual linear translation stage and two pin headers. Related to Figure 4.

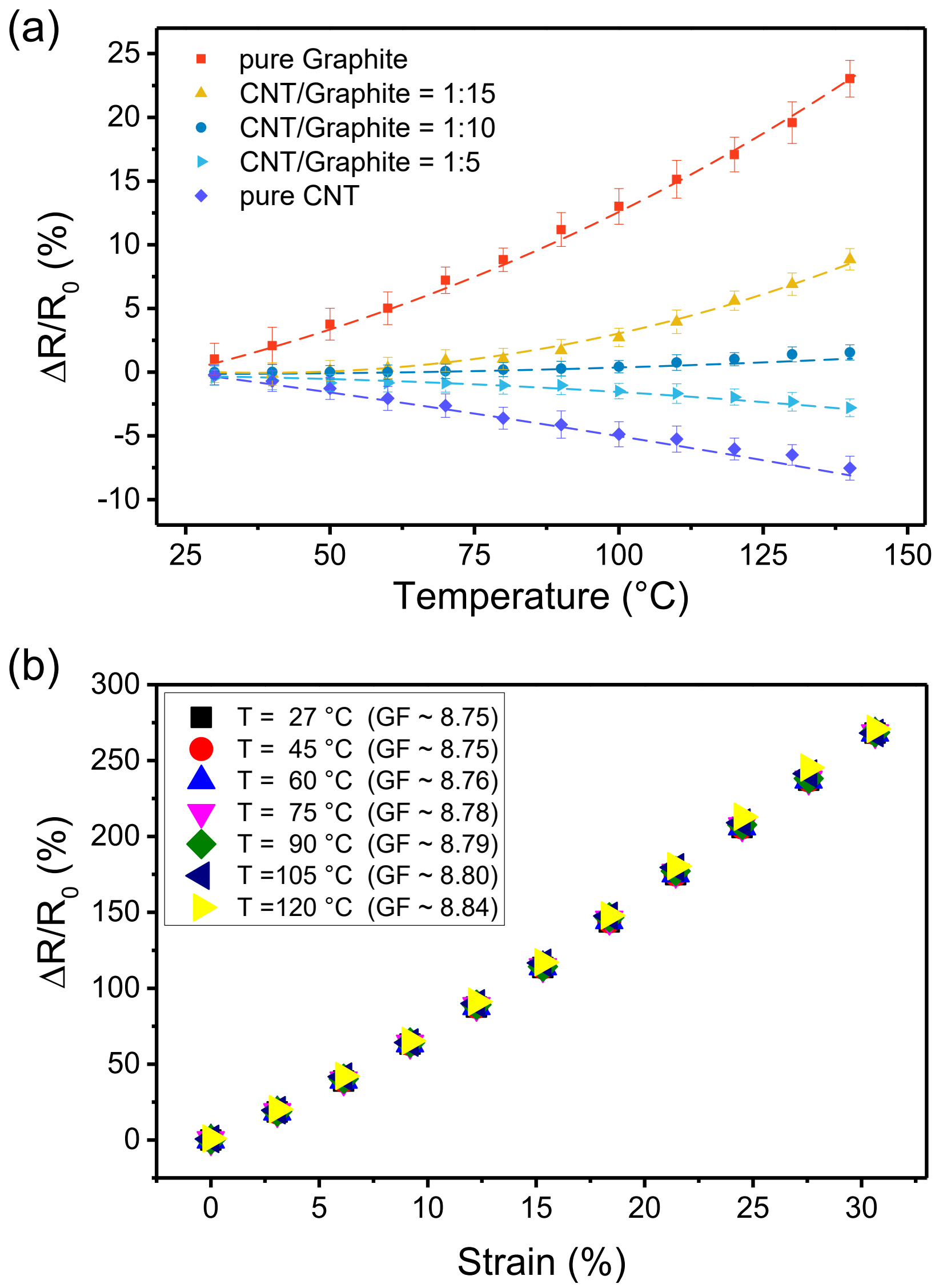

Figure S2. **(a)**Temperature dependence of relative resistance change of the CCL with different CNT–graphite ratios. The results indicate that pure graphite exhibits a strong positive TCR, while pure CNT exhibits a strong negative TCR. **(b)** Relationships between relative resistance change and strain for a sample with a mass ratio of 1:10 at different temperatures. The responses remain highly linear over a tensile strain range of 0%–30%. Data are represented as mean (n = 10), with error bars indicating the minimum-to-maximum range. Related to Figure 5.

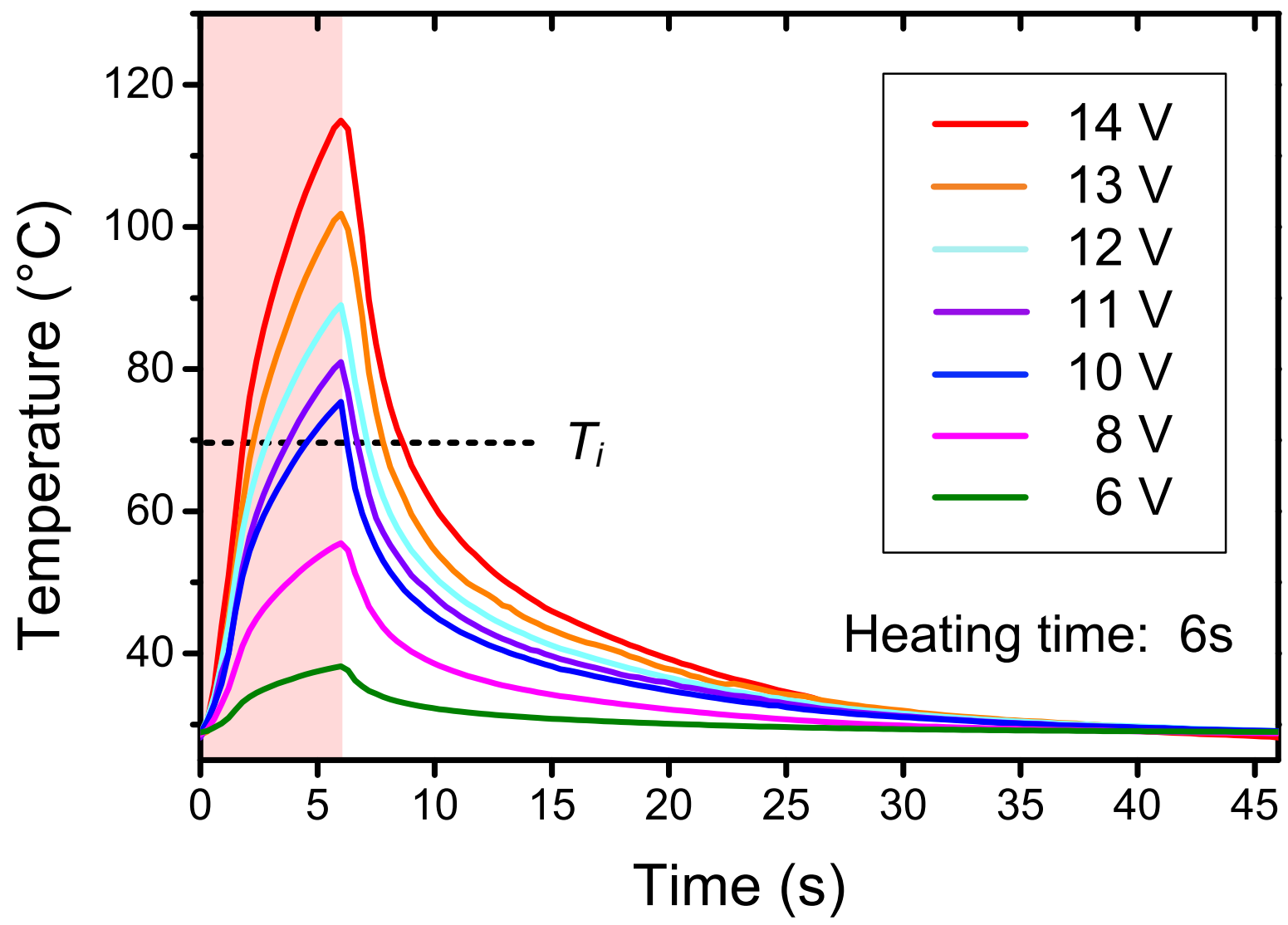

Figure S3. Transient temperature response with a fixed heating time (6 s) at different actuating voltages. Supplying voltages of 10 V or greater can rapidly increase the temperature beyond 70 °C within 6 s. Consequently, elevating the actuation voltage effectively reduces the time required to reach the phase transition temperature. Related to Figure 5.

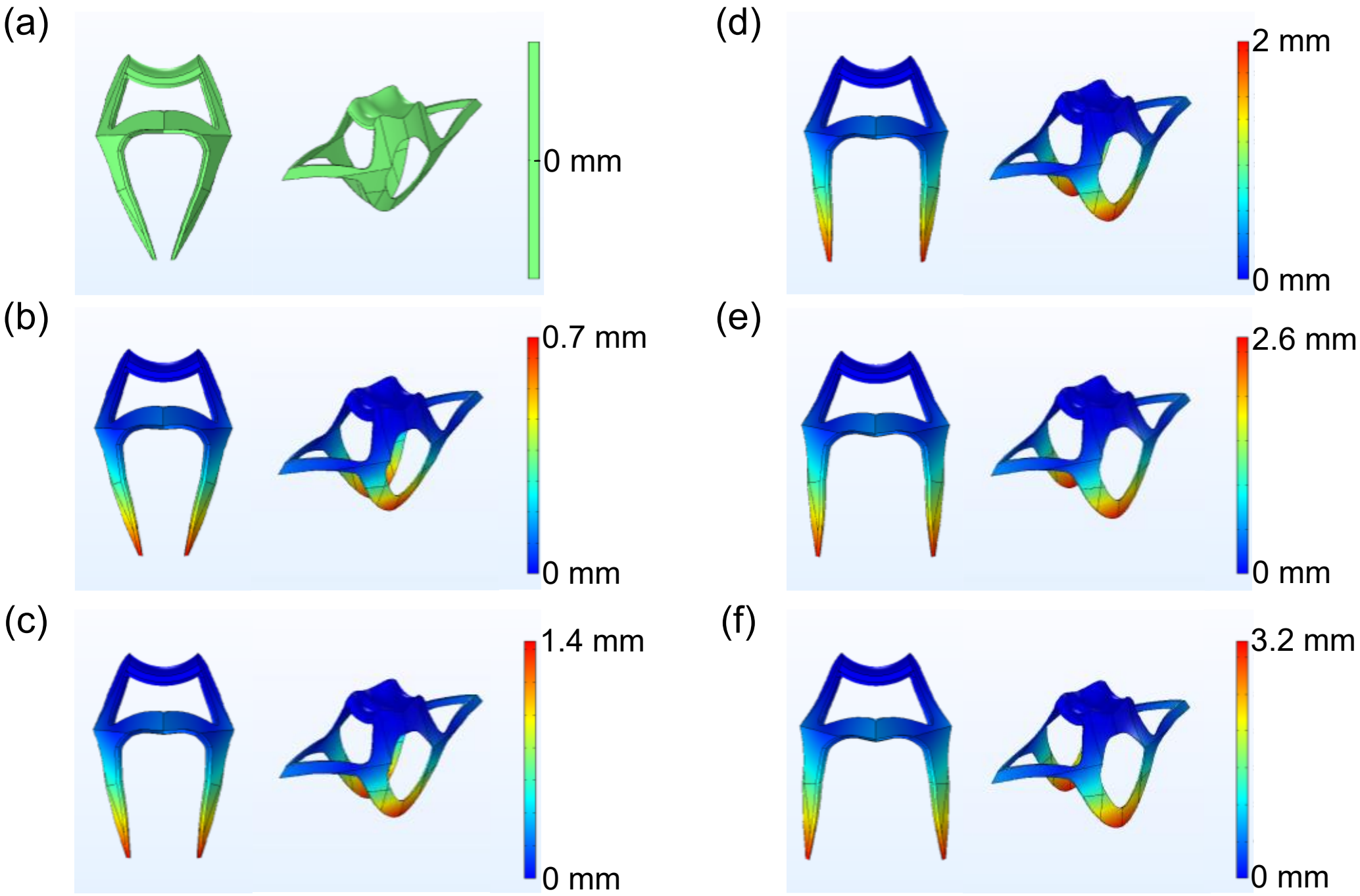

Figure S4. Finite Element Analysis (FEA) simulation of the tip displacement of the gripper structure under different applied forces on finger-tip regions: from (a) to (f), the applied force increases from 0 μN to 37.5 μN, with an increment of 7.5 μN. Related to Figure 7.

Table S1. Physical parameters used in the FEM simulation. Related to STAR methods.

Parameter	Materials	
	LCE	Conductive composite (for thermal analysis only)
Density [kg/m^3]	970	1400
Young's modulus [kPa]	750	-
Poisson's ratio	0.49	-
Thermal conductivity [W/(m·k)]	0.16	3000
Heat capacity at constant pressure [J/(kg·K)]	1460	700
Electrical conductivity [S/m]	-	100000
